# Identification of *In Vitro* Inhibitors of Monkeypox Replication

**DOI:** 10.1128/spectrum.04745-22

**Published:** 2023-06-06

**Authors:** Kevin Chiem, Aitor Nogales, Maria Lorenzo, Desarey Morales Vasquez, Yan Xiang, Yogesh K. Gupta, Rafael Blasco, Juan Carlos de la Torre, Luis Martínez-Sobrido

**Affiliations:** a Texas Biomedical Research Institute, San Antonio, Texas, USA; b Animal Health Research Centre, Centro Nacional Instituto de Investigación y Tecnología Agraria y Alimentaria, Madrid, Spain; c Departamento de Biotecnología, Instituto Nacional de Investigación y Tecnología Agraria y Alimentaria, Madrid, Spain; d Department of Microbiology, Immunology & Molecular Genetics, University of Texas Health Science Center at San Antonio, San Antonio, Texas, USA; e Department of Biochemistry and Structural Biology, University of Texas Health Science Center at San Antonio, San Antonio, Texas, USA; f Department of Immunology and Microbiology, The Scripps Research Institute, La Jolla, California, USA; IrsiCaixa Institut de Recerca de la Sida

**Keywords:** antivirals, GFP, luciferase, monkeypox, orthopoxvirus, poxvirus, mScarlet, vaccinia virus

## Abstract

Monkeypox virus (MPXV) infections in humans have historically been restricted to regions of endemicity in Africa. However, in 2022, an alarming number of MPXV cases were reported globally, with evidence of person-to-person transmission. Because of this, the World Health Organization (WHO) declared the MPXV outbreak a public health emergency of international concern. The supply of MPXV vaccines is limited, and only two antivirals, tecovirimat and brincidofovir, approved by the U.S. Food and Drug Administration (FDA) for the treatment of smallpox, are currently available for the treatment of MPXV infection. Here, we evaluated 19 compounds previously shown to inhibit different RNA viruses for their ability to inhibit orthopoxvirus infections. We first used recombinant vaccinia virus (rVACV) expressing fluorescence (mScarlet or green fluorescent protein [GFP]) and luciferase (Nluc) reporter genes to identify compounds with antiorthopoxvirus activity. Seven compounds from the ReFRAME library (antimycin A, mycophenolic acid, AVN-944, pyrazofurin, mycophenolate mofetil, azaribine, and brequinar) and six compounds from the NPC library (buparvaquone, valinomycin, narasin, monensin, rotenone, and mubritinib) showed inhibitory activity against rVACV. Notably, the anti-VACV activity of some of the compounds in the ReFRAME library (antimycin A, mycophenolic acid, AVN-944, mycophenolate mofetil, and brequinar) and all the compounds from the NPC library (buparvaquone, valinomycin, narasin, monensin, rotenone, and mubritinib) were confirmed with MPXV, demonstrating their inhibitory activity *in vitro* against two orthopoxviruses.

**IMPORTANCE** Despite the eradication of smallpox, some orthopoxviruses remain important human pathogens, as exemplified by the recent 2022 monkeypox virus (MPXV) outbreak. Although smallpox vaccines are effective against MPXV, access to those vaccines is limited. In addition, current antiviral treatment against MPXV infections is limited to the use of the FDA-approved drugs tecovirimat and brincidofovir. Thus, there is an urgent need to identify novel antivirals for the treatment of MPXV infection and other potentially zoonotic orthopoxvirus infections. Here, we show that 13 compounds, derived from two different libraries, previously found to inhibit several RNA viruses, also inhibit VACV. Notably, 11 compounds also displayed inhibitory activity against MPXV.

## INTRODUCTION

In 2022, the World Health Organization (WHO) declared the monkeypox virus (MPXV) outbreak a public health emergency of international concern (1–3). Historically, MPXV infections in humans have rarely been reported outside its regions of endemicity in Africa (4), but in 2022, MPXV cases were reported worldwide, with sustained person-to-person transmission (1). Most of the confirmed cases in regions where the disease is not endemic are in Europe and North America (2), with no clear epidemiological links to countries where it is endemic (5–7). As of March 2023, over 30,286 confirmed cases and 38 deaths related to MPXV infections have been reported in the United States (8).

MPXV belongs to the genus *Orthopoxvirus* of the poxvirus family, which includes variola virus (VARV), cowpox virus, and vaccinia virus (VACV) (9, 10). Poxviruses are large double-stranded DNA viruses with genomes ranging from 135 to 380 kb encoding up to 328 predicted open reading frames (ORFs) (11). Currently, two clades of MPXV have been recognized: clade I, predominant in the Congo Basin and responsible for up to 10% lethality in humans; clade IIa, found in West Africa and responsible for low mortality in humans; and clade IIb, responsible for the current global spread in humans (12). To date, all MPXV cases associated with the 2022 global outbreak appear to be related to clade II (13, 14). MPXV was first discovered in research monkeys in an animal facility in Copenhagen, Denmark, that had been shipped from Singapore in 1958 (15, 16). Despite its name, the natural reservoir of MPXV clades I and IIa is not nonhuman primates, and instead, it has been speculated to be small rodents native to Central and West Africa, respectively (17–21). Zoonotic transmission of MPXV most likely is mediated by body fluids, such as salivary or respiratory droplets, or derived from wounds (9, 22, 23). Person-to-person transmission of MPXV occurs by prolonged close contact, for instance, prolonged face-to-face or intimate physical contact, touching of infectious lesions or bodily fluids, and contact with contaminated fomites (23–25).

Currently, two vaccines are available for the prophylactic treatment of MPXV infection, a modified vaccinia Ankara (MVA; JYNNEOS in the United States, IMVANEX in the European Union, and IMAMUNE in Canada) (26–33) and ACAM2000 (34–36); the latter is available for use under an Expanded Access Investigational New Drug (EA-IND) protocol. However, vaccine supplies are limited and are reserved for individuals that are at high risk, including immunocompromised individuals and men that are sexually active with men (37). The only available FDA-approved treatments against poxviruses are tecovirimat and brincidofovir (38–41). Tecovirimat has been shown to target the VP37 protein (the product of the F13L gene) of VACV, which is required for extracellular virus particle formation and is highly conserved in the genus *Orthopoxvirus* (42, 43). However, resistance mutations to tecovirimat have been reported for various orthopoxviruses, including VACV, camelpox virus, and cowpox virus (38, 44). Due to the severity of the MPXV outbreak, brincidofovir is available under the EA-IND protocol (45), but its efficacy has not been fully defined outside animal models (46–53). Therefore, the importance of developing new therapeutics targeting host factors or virus-host interactions to combat *Orthopoxvirus* infections cannot be underestimated. The discovery and implementation of new antivirals constitute a labor-intensive and costly process requiring multiple rounds of testing and refinement to ensure their safety and efficacy. Repurposing current FDA-approved drugs can facilitate the advancement of candidate antiviral drugs into the clinic, since the pharmacology and toxicology of the drug have already been established.

In this study, we used a previously described bireporter cell-based assay based on the use of recombinant vaccinia virus (rVACV) expressing fluorescent proteins (green fluorescent protein [GFP] or mScarlet [Scarlet]) and luciferase (Nluc) (54) to assess the antiviral efficacy of compounds previously identified as having inhibitory activity against different RNA viruses. These compounds belonged to two libraries: Repurposing, Focused Rescue, and Accelerated Medchem (ReFRAME) (55–58) and NCATS Pharmaceutical Collection (NPC) (59). We found that seven of the compounds from the ReFRAME library (antimycin A, mycophenolic acid, AVN-944, pyrazofurin, mycophenolate mofetil, azaribine, and brequinar) and six compounds from the NPC library (buparvaquone, valinomycin, narasin, monensin, rotenone, and mubritinib) had inhibitory activity against VACV. Importantly, some of the identified compounds also inhibit MPXV.

## RESULTS

### A bireporter cell-based assay for the identification of VACV inhibitors.

**FIG 1:**
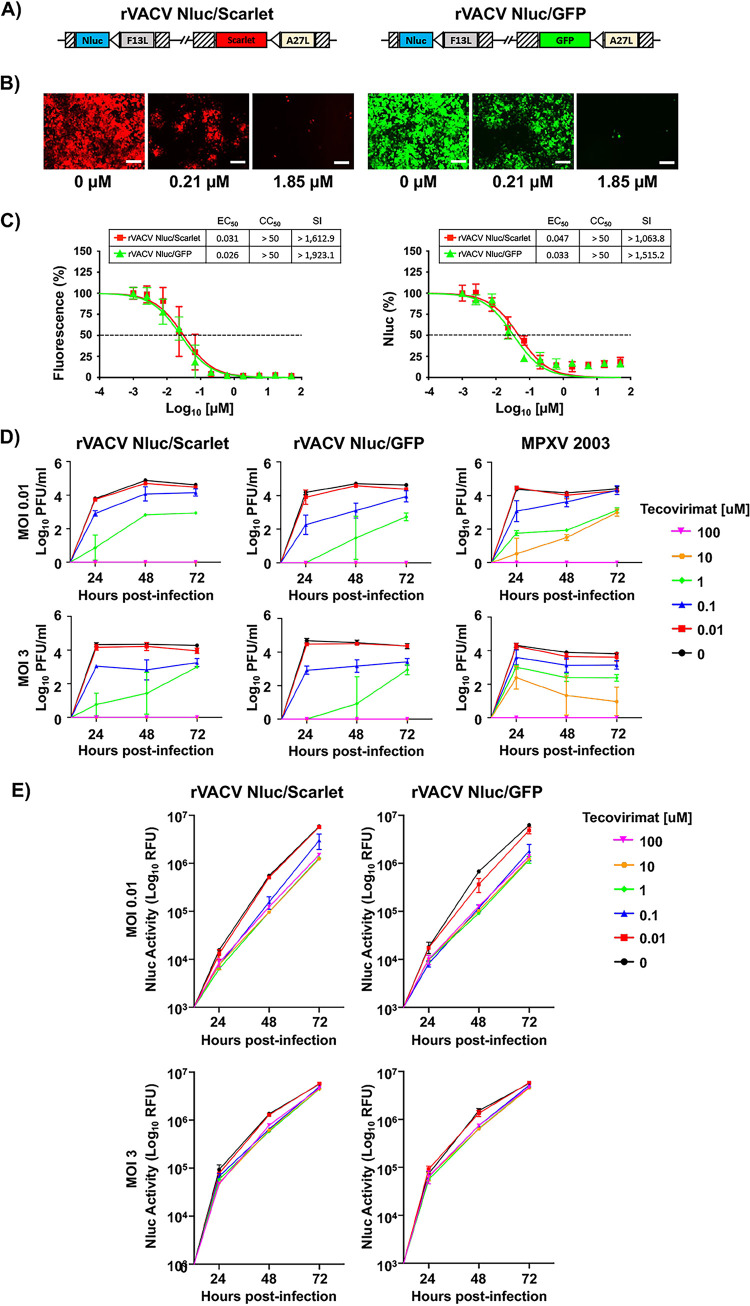
Bireporter cell-based assay for the identification of VACV inhibitors. (A) Schematic representation of rVACV Nluc/Scarlet (left) and rVACV Nluc/GFP (right). Nluc (blue boxes) and fluorescent reporter Scarlet (red boxes) or GFP (green boxes) genes were inserted downstream of F13L (gray boxes) or A27L (yellow boxes), respectively, in the viral genome. The reporter genes are expressed by an early/late VACV synthetic promoter (white arrows). Hatched boxes indicate the recombination flanking regions for the insertion of reporter genes into the VACV genome. (B and C) Bireporter cell-based assay to identify inhibitors. Human A549 cells (2 × 10^4^ cells/well, 96-well plates, in quadruplicate) were infected with 200 PFU of rVACV Nluc/Scarlet (left) or rVACV Nluc/GFP (right) and incubated with 3-fold serial dilutions (starting concentration of 50 μM) of tecovirimat. Mock-infected cells and cells infected in the absence of drug were included as internal controls. At 24 hpi, Scarlet (rVACV Nluc/Scarlet, left) and GFP (rVACV Nluc/GFP, right) expression was visualized under a fluorescence microscope (B). Representative images are shown. Bars, 100 μm. Magnification, ×20. To quantify inhibition of viral replication, Scarlet (red squares) or GFP (green triangles) expression levels were quantified at 24 hpi using a fluorescent microplate reader (C, left) or Nluc expression (C, right) was assessed using a luminometer for rVACV Nluc/Scarlet (red squares) or rVACV Nluc/GFP (green triangles). The EC_50_ of tecovirimat was calculated using sigmoidal dose-response curves. The CC_50_ of tecovirimat was determined using an MTT assay kit. The SI was calculated by dividing the CC_50_ by the EC_50_. The percent viral inhibition was normalized to non-tecovirimat-treated controls. The dotted lines indicate 50% inhibition. Data are means and SD of viral inhibition from quadruplicate wells (*n* = 4). (D and E) Viral titers (D) and Nluc activity (E). Human A549 cells (2 × 10^4^ cells/well, 96-well plates, in triplicate) were infected with an MOI of 0.01 (top) or 3 (bottom) of rVACV Nluc/Scarlet, rVACV Nluc/GFP, or MPXV for 1 h and incubated with 10-fold serial dilutions (starting concentration of 100 μM) of tecovirimat. At 24, 48, and 72 hpi, tissue culture supernatants were collected, and viral titers were determined by plaque assay in CV1 cells. Additionally, Nluc activity (E) in cell culture supernatants was determined using a luminometer for rVACV Nluc/Scarlet (left) or rVACV Nluc/GFP (right). Mock-infected cells and cells infected in the absence of drug were included as internal controls. Data are means and SD of viral inhibition from triplicate wells (*n* = 3).

To determine the inhibitory activity of compounds from the ReFRAME (55–57) and NPC (59) libraries against orthopoxviruses, which were identified as having antiviral activity against different RNA viruses, we used our previously described fluorescent protein (Scarlet or GFP)- and Nluc-expressing rVACV (rVACV Nluc/Scarlet and rVACV Nluc/GFP) (Fig. 1A) (54). We have shown that expression levels of reporter genes from these rVACVs can be used as an accurate surrogate for viral infection (54). First, we validated the use of reporter-expressing rVACV (rVACV Nluc/Scarlet and rVACV Nluc/GFP) by assessing the inhibitory activity of tecovirimat, an FDA-approved antiviral against poxviruses (38–41). We used 3-fold serial dilutions (starting at 50 μM) of tecovirimat to treat human A549 cells infected with rVACV Nluc/Scarlet or rVACV Nluc/GFP, and viral infection was assessed based on fluorescent and luciferase expression (Fig. 1B and C). Tecovirimat exhibited a dose-dependent inhibitory effect in Scarlet (Fig. 1B, left) or GFP (right) expression levels. Tecovirimat half-maximal effective concentrations (EC_50_s) for rVACV Nluc/Scarlet and Nluc/GFP were similar, 0.031 and 0.026 μM, respectively, regardless of which fluorescence reporter was used for quantifications (Fig. 1C, left). Likewise, EC_50_s calculated by assessing Nluc activity in the cell culture supernatants from rVACV Nluc/Scarlet- and rVACV Nluc/GFP-infected cells were also similar (0.047 and 0.033 μM, respectively) (Fig. 1C, right). Selectivity index (SI) values based on Scarlet or GFP levels were also similar (>1,612.9 and >1,923.1, respectively) and comparable to those based on Nluc from either rVACV Nluc/Scarlet or rVACV Nluc/GFP (>1,063.8 and >1,515.2, respectively). To further validate the use of bireporter rVACV to assess compound inhibitory activity, we conducted a viral titer reduction assay. For this we infected A549 cells at low (0.01) and high (3) multiplicities of infection (MOI) and treated them with 10-fold serial dilutions of tecovirimat (starting concentration of 100 μM). Cell culture supernatants were collected at 24, 48, and 72 hpi. Then, extracellular viral titers and Nluc activity (rVACV Nluc/Scarlet or rVACV Nluc/GFP) were determined from the cell culture supernatants. We found a dose-dependent decrease in extracellular viral titers of rVACV Nluc/Scarlet, rVACV Nluc/GFP, and MPXV (Fig. 1D). Tecovirimat acts primarily by inhibiting the activity of VACV protein VP37, which is required for virus egress. This results in decreased production of extracellular virus and viral spread without affecting gene expression, reflected in cell culture supernatants having Nluc activity even in the presence of the highest concentration (100 μM) of tecovirimat (Fig. 1E). The decrease in extracellular viral titers in high-MOI infections in the presence of tecovirimat presumably reflects inhibition of extracellular virus formation by the drug. Nonetheless, the antiviral activities of tecovirimat obtained with rVACV Nluc/Scarlet and rVACV Nluc/GFP and with MPXV were consistent with those reported in the literature for VACV (60) and MPXV (38, 39, 43, 61, 62). These results demonstrated that rVACV Nluc/Scarlet or rVACV Nluc/GFP can be used to determine accurately the inhibitory properties of compounds against orthopoxvirus based on fluorescent or luciferase expression.

### Effect of selected compounds from the ReFRAME and NPC libraries on VACV multiplication.

**FIG 2:**
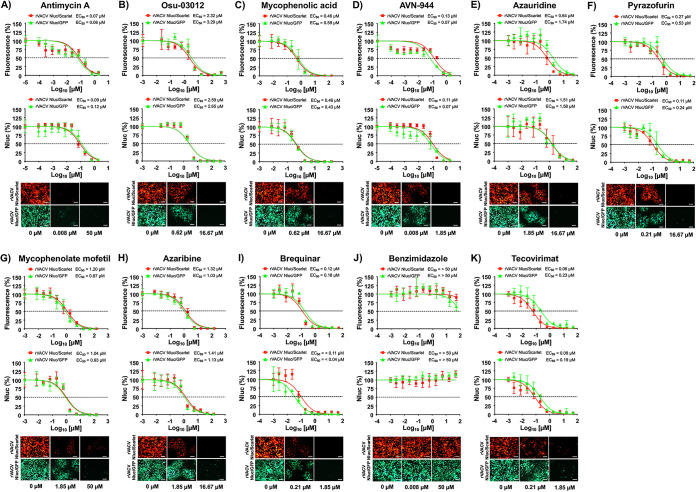
Inhibitory activity of the ReFRAME compounds against VACV: Human A549 cells (2 × 10^4^ cells/well, 96-well plates, in quadruplicate) were infected with 200 PFU of rVACV Nluc/Scarlet (red squares) or rVACV Nluc/GFP (green triangles) and incubated with 3-fold serial dilutions of antimycin A (A), Osu-03012 (B), mycophenolic acid (C), AVN-944 (D), azauridine (E), pyrazofurin (F), mycophenolate mofetil (G), azaribine (H), or brequinar (I). Benzimidazole (J) and tecovirimat (K) were included as negative and positive controls, respectively. Mock-infected cells and cells infected in the absence of drug were included as internal controls. At 24 hpi, inhibition of rVACV Nluc/Scarlet and rVACV Nluc/GFP viral replication was evaluated by quantifying fluorescent Scarlet or GFP (top graphs) and Nluc (bottom graphs) expression using a fluorescent microplate reader and a luminometer, respectively. The EC_50_ for each compound was calculated using sigmoidal dose-response curves with GraphPad Prism. The dotted lines indicate 50% inhibition. Data are means and SD of viral inhibition from quadruplicate wells (*n* = 4). At the same time point (hpi), Scarlet (rVACV Nluc/Scarlet, top) and GFP (rVACV Nluc/GFP, bottom) fluorescent expression in infected cells in the absence (0 μM) or in the presence of the indicated concentrations of the compounds (maximum concentration and EC_50_) was visualized using a fluorescence microscope. Representative images are shown. Bars, 100 μm. Magnification, ×20.

**FIG 3:**
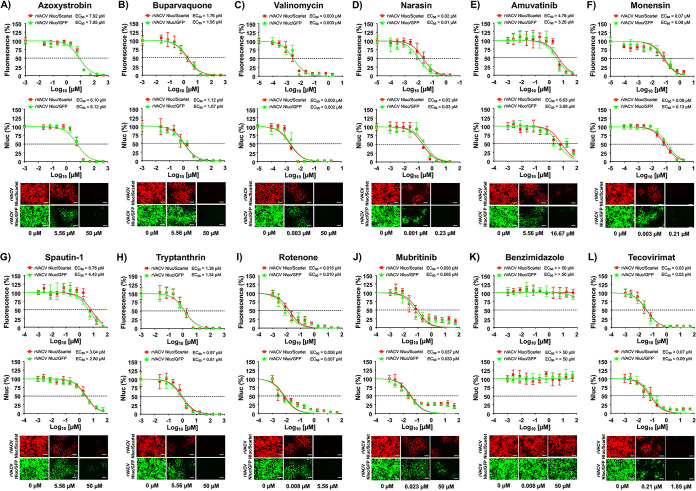
Inhibitory activity of the NPC compounds against VACV. Human A549 cells (2 × 10^4^ cells/well, 96-well plates, in quadruplicate) were infected with 200 PFU of rVACV Nluc/Scarlet (red squares) or rVACV Nluc/GFP (green triangles) and incubated with 3-fold serial dilutions of azoxystrobin (A), buparvaquone (B), valinomycin (C), narasin (D), amuvatinib (E), monensin (F), spautin-1 (G), tryptanthrin (H), rotenone (I), or mubritinib (J). Benzimidazole (K) and tecovirimat (L) were included as negative and positive controls, respectively. Mock-infected cells and cells infected in the absence of drug were included as internal controls. Inhibition of rVACV Nluc/Scarlet or rVACV Nluc/GFP viral replication was evaluated by quantifying Scarlet or GFP (top graphs) and Nluc (bottom graphs) expression at 24 hpi using fluorescent and luciferase microplate readers, respectively. The EC_50_ for each compound was calculated using sigmoidal dose-response curves with GraphPad Prism. The dotted lines indicate 50% inhibition. Data are means and SD of viral inhibition from quadruplicate wells (*n* = 4). At the same time point (hpi), Scarlet (rVACV Nluc/Scarlet; top) and GFP (rVACV Nluc/GFP; bottom) fluorescent expression in infected cells in the absence (0 μM) or presence of the indicated concentrations of the compounds (maximum concentration and EC_50_) was visualized using a fluorescence microscope. Representative images are shown. Bars, 100 μm. Magnification, ×20.

To assess whether ReFRAME and NPC compounds previously identified to have antiviral activity against arenaviruses, influenza A and B viruses (IAV and IBV, respectively), and Zika virus (ZIKV) (55–59) also inhibited poxvirus infection, we used the above-described bireporter cell-based assay based on the use of rVACV Nluc/Scarlet and rVACV Nluc/GFP. We included benzimidazole and tecovirimat as negative and positive controls, respectively. Seven of the compounds tested from the ReFRAME library showed potent inhibition of rVACV Nluc/Scarlet and rVACV Nluc/GFP based on fluorescence (Fig. 2A to I, top) and Nluc (Fig. 2A to I, bottom) activities (Table 1). As expected, we did not observe any inhibitory activity in cells treated with benzimidazole (Fig. 2J and Table 1), while tecovirimat antiviral activity (Fig. 2K and Table 1) was consistent with the results of our validation experiment (Fig. 1) and those reported in the literature (38, 39, 43, 61, 62). Antimycin A showed potent inhibitory activity based on fluorescence (EC_50_ Scarlet = 0.07 μM; EC_50_ GFP = 0.06 μM) or Nluc (EC_50_ Scarlet = 0.09 μM; EC_50_ GFP = 0.12 μM) expression. However, the SI value of antimycin A differed based on the median cytotoxic concentration (CC_50_) results from the MTT [3-(4,5-dimethyl-2-thiazolyl)-2,5-diphenyl-2H-tetrazolium bromide] and XTT [2,3-bis-(2-methoxy-4-nitro-5-sulfophenyl)-2H-tetrazolium-5-carboxanilide salt] toxicity assays (Table 1). AVN-944 also exhibited potent inhibitory properties against VACV, as determined by fluorescence (Scarlet EC_50_ = 0.13 μM; GFP EC_50_ = 0.07 μM) or Nluc (Scarlet EC_50_ = 0.11 μM; GFP EC_50_ = 0.07 μM) expression. In the case of AVN-944, the SI value was not altered by the CC_50_ results from the MTT and XTT toxicity assays (Table 1). Other compounds from the ReFRAME library also showed inhibition of rVACV, including mycophenolic acid, pyrazofurin, mycophenolate mofetil, azaribine, and brequinar (Fig. 2 and Table 1). Likewise, six compounds from the NPC library (buparvaquone, valinomycin, narasin, monensin, rotenone, and mubritinib), previously found to have antiarenaviral activity (59), showed potent inhibition of rVACV Nluc/Scarlet and rVACV Nluc/GFP based on fluorescence (Fig. 3A to J, top) and Nluc (Fig. 3A to J, bottom) expression (Table 2). As described above, benzimidazole (Fig. 3J and Table 2) and tecovirimat (Fig. 3K and Table 2) treatment had no and potent, respectively, antiviral activity. The most potent inhibitors against VACV in the NPC library were valinomycin, rotenone, and mubritinib, followed by monensin, narasin, and buparvaquone (Table 2). In the case of the compounds from the NPC library, the SI values, except that for monensin, were different based on the CC_50_ results from the MTT and XTT toxicity assays (Table 2). These results demonstrated that compounds in the ReFRAME (Fig. 2 and Table 1) and NPC (Fig. 3 and Table 2) libraries previously identified as having antiviral activity against different RNA viruses (55–57, 59) also have potent inhibitory activity against VACV, demonstrating the ability of these compounds to inhibit different RNA and DNA viruses.

**TABLE 1:** CC_50_, EC_50_, and SI of the ReFRAME antiviral compounds against the bireporter rVACV^*a*^

Compound	Virus	CC_50_ (μM)		EC_50_ (μM)		SI			
						FP		Nluc	
		MTT	XTT	FP	Nluc	MTT	XTT	MTT	XTT
Antimycin A	rVACV Scarlet/Nluc	8.73	39.29	0.07	0.09	125	561	97	437
	rVACV GFP/Nluc	8.73	39.29	0.06	0.12	146	655	73	327
Osu-03012	rVACV Scarlet/Nluc	22.23	43.72	2.32	2.59	10	19	9	17
	rVACV GFP/Nluc	22.23	43.72	3.29	2.65	7	13	8	16
Mycophenolic acid	rVACV Scarlet/Nluc	>150	>150	0.46	0.46	>326	>326	>326	>326
	rVACV GFP/Nluc	>150	>150	0.59	0.43	>254	>254	>349	>349
AVN-944	rVACV Scarlet/Nluc	>50	>50	0.13	0.11	>385	>385	>455	>455
	rVACV GFP/Nluc	>50	>50	0.07	0.07	>714	>714	>714	>714
Azauridine	rVACV Scarlet/Nluc	>50	>50	0.64	1.51	>78	>78	>33	>33
	rVACV GFP/Nluc	>50	>50	1.74	1.58	>29	>29	>32	>32
Pyrazofurin	rVACV Scarlet/Nluc	>50	>50	0.27	0.11	>185	>185	>455	>455
	rVACV GFP/Nluc	>50	>50	0.53	0.24	>94	>94	>208	>208
Mycophenolate mofetil	rVACV Scarlet/Nluc	>150	>150	1.20	1.04	>125	>125	>144	>144
	rVACV GFP/Nluc	>150	>150	0.87	0.93	>172	>172	>161	>161
Azaribine	rVACV Scarlet/Nluc	>150	>150	1.32	1.41	>114	>114	>106	>106
	rVACV GFP/Nluc	>150	>150	1.03	1.13	>146	>146	>133	>133
Brequinar	rVACV Scarlet/Nluc	>50	>50	0.12	0.11	>417	>417	>455	>455
	rVACV GFP/Nluc	>50	>50	0.18	0.04	>278	>278	>1,250	>1,250
Benzimidazole	rVACV Scarlet/Nluc	>50	>50	>50	>50	NA	NA	NA	NA
	rVACV GFP/Nluc	>50	>50	>50	>50	NA	NA	NA	NA
Tecovirimat	rVACV Scarlet/Nluc	>50	>50	0.06	0.08	>833	>833	>625	>625
	rVACV GFP/Nluc	>50	>50	0.23	0.19	>217	>217	>263	>263

a FP, fluorescent protein; NA, not applicable.

**TABLE 2:** CC_50_, EC_50_, and SI of the NPC antiviral compounds against the bireporter rVACV^*a*^

Compound	Virus	CC_50_ (μM)		EC_50_ (μM)		SI			
						FP		Nluc	
		MTT	XTT	FP	Nluc	MTT	XTT	MTT	XTT
Azoxystrobin	rVACV Scarlet/Nluc	27.92	>450	7.92	6.10	4	>57	5	>74
	rVACV GFP/Nluc	27.92	>450	7.85	6.12	4	>57	5	>74
Buparvaquone	rVACV Scarlet/Nluc	121.4	>450	1.76	1.12	69	>256	108	>402
	rVACV GFP/Nluc	121.4	>450	1.95	1.07	62	>231	113	>421
Valinomycin	rVACV Scarlet/Nluc	0.29	13.63	0.003	0.002	97	4,543	145	6,815
	rVACV GFP/Nluc	0.29	13.63	0.003	0.002	97	4,543	145	6,815
Narasin	rVACV Scarlet/Nluc	1.3	8.73	0.02	0.02	65	437	65	437
	rVACV GFP/Nluc	1.3	8.73	0.01	0.03	130	873	43	291
Amuvatinib	rVACV Scarlet/Nluc	18.51	1.80	4.76	6.63	4	0.4	3	0.3
	rVACV GFP/Nluc	18.51	1.80	3.26	3.89	6	1	5	0.5
Monensin	rVACV Scarlet/Nluc	>50	>50	0.07	0.08	>714	>714	>625	>625
	rVACV GFP/Nluc	>50	>50	0.08	0.13	>625	>625	>385	>385
Spautin-1	rVACV Scarlet/Nluc	8.00	>50	6.78	3.04	1	>7	3	>16
	rVACV GFP/Nluc	8.00	>50	4.43	2.80	2	>11	3	>18
Tryptanthrin	rVACV Scarlet/Nluc	6.31	16.65	1.38	0.97	5	12	7	17
	rVACV GFP/Nluc	6.31	16.65	1.34	0.81	5	12	8	21
Rotenone	rVACV Scarlet/Nluc	0.10	>50	0.015	0.008	7	>3,333	13	>6,250
	rVACV GFP/Nluc	0.10	>50	0.010	0.007	10	>5,000	14	>7,143
Mubritinib	rVACV Scarlet/Nluc	0.07	>50	0.093	0.037	1	>538	2	>1,351
	rVACV GFP/Nluc	0.07	>50	0.065	0.033	1	>769	2	>1,515
Benzimidazole	rVACV Scarlet/Nluc	>50	>50	>50	>50	NA	NA	NA	NA
	rVACV GFP/Nluc	>50	>50	>50	>50	NA	NA	NA	NA
Tecovirimat	rVACV Scarlet/Nluc	>50	>50	0.03	0.07	>1,667	>1,667	>714	>714
	rVACV GFP/Nluc	>50	>50	0.03	0.09	>1,667	>1,667	>556	>556

a FP, fluorescent protein; NA, not applicable.

### Effect of selected compounds from the ReFRAME and NPC libraries on MPXV multiplication.

**FIG 4:**
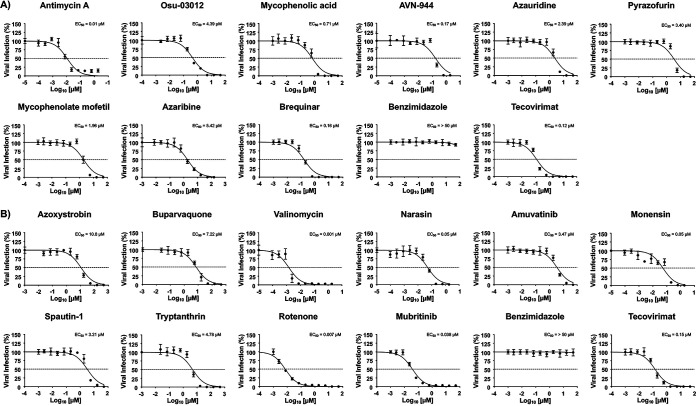
Inhibitory activity of the ReFRAME and NPC compounds against MPXV. Human A549 cells (2 × 10^4^ cells/well, 96-well plates, in quadruplicate) were infected with 200 PFU of MPXV and incubated with 3-fold serial dilutions (starting concentration of 50 μM) of the ReFRAME library compound antimycin A, Osu-03012, mycophenolic acid, AVN-944, azauridine, pyrazofurin, mycophenolate mofetil, azaribine, or brequinar (A) or the NPC library compound azoxystrobin, buparvaquone, valinomycin, narasin, amuvatinib, monensin, spautin-1, tryptanthrin, rotenone, or mubritinib (B). Benzimidazole and tecovirimat were included as negative and positive controls, respectively (A and B). Mock-infected cells and cells infected in the absence of drug were included as an internal control. Inhibition of viral replication was evaluated by quantifying the number of plaques at 24 hpi using a cross-reactive anti-VACV A33R polyclonal antibody and developed with an anti-rabbit Vectastain and DAB reagent. The EC_50_ of each compound was calculated using sigmoidal dose-response curves with GraphPad Prism. The dotted line indicates 50% inhibition. Data are means and SD of viral inhibition from quadruplicate wells (*n* = 4).

VACV and MPXV are both orthopoxviruses and share similar genetic and biological properties. We therefore examined whether compounds with anti-VACV activity from the ReFRAME and NPC libraries were also able to inhibit MPXV infection. To assess the ability of the compounds in the ReFRAME and NPC libraries to inhibit MPXV, we used a focus-forming reduction assay (FFRA). We found that five of the compounds tested from the ReFRAME library (Fig. 4A and Table 3) and all of the compounds from the NPC library (Fig. 4B and Table 4) also had inhibitory activity against MPXV. As anticipated, benzimidazole (Fig. 4A and B) did not inhibit MPXV infection, whereas tecovirimat (Fig. 4A and B) potently inhibited MPXV (63). As with VACV, antimycin A, AVN-944, and brequinar from the ReFRAME library (Table 3) and valinomycin, rotenone, and mubritinib from the NPC library (Table 4) were the compounds with the strongest inhibitory activity. However, similar to the results with VACV, the SI values of the compounds differed based on the CC_50_ values obtained from the MTT and XTT toxicity assays (Tables 3 and 4). These results demonstrated that a set of compounds from the ReFRAME and NPC libraries we previously identified as having antiviral activity against RNA viruses also inhibit the DNA viruses VACV and MPXV, expanding their potential as inhibitors against a diverse collection of RNA and DNA viruses.

**TABLE 3:** CC_50_, EC_50_, and SI of the ReFRAME antiviral compounds against MPXV^*a*^

Compound	CC_50_ (μM)		EC_50_ (μM)	SI	
	MTT	XTT		MTT	XTT
Antimycin A	8.73	39.29	0.01	873	3,929
Osu-03012	22.23	43.72	4.39	5	10
Mycophenolic acid	>150	>150	0.71	>211	>211
AVN-944	>50	>50	0.17	>294	>294
Azauridine	>50	>50	2.39	>21	>21
Pyrazofurin	>50	>50	3.40	>15	>15
Mycophenolate mofetil	>150	>150	1.96	>77	>77
Azaribine	>150	>150	5.42	>28	>28
Brequinar	>50	>50	0.16	>313	>313
Benzimidazole	>50	>50	>50	NA	NA
Tecovirimat	>50	>50	0.12	>417	>417

a NA, not applicable.

**TABLE 4:** CC_50_, EC_50_, and SI of the NPC antiviral compounds against MPXV^*a*^

Compound	CC_50_ (μM)		EC_50_ (μM)	SI	
	MTT	XTT		MTT	XTT
Azoxystrobin	27.92	>450	10.8	3	>42
Buparvaquone	121.4	>450	7.22	17	>62
Valinomycin	0.29	13.63	0.001	290	13,630
Narasin	1.3	8.73	0.05	26	175
Amuvatinib	18.51	1.8	3.47	5	1
Monensin	>50	>50	0.05	>1,000	>1,000
Spautin-1	8.00	>50	3.21	2	>16
Tryptanthrin	6.31	16.65	4.78	1	3
Rotenone	0.10	>50	0.007	14	>7,143
Mubritinib	0.07	>50	0.038	2	>1,316
Benzimidazole	>50	>50	>50	NA	NA
Tecovirimat	>50	>50	0.15	>333	>333

a NA, not applicable.

## DISCUSSION

The MPXV outbreak in early 2022 was declared a public health emergency of international concern by the WHO, with over 30,286 confirmed cases and 38 deaths in the United States as of March 2023 (86,746 cases and 112 deaths worldwide) (1–3). Alarmingly, the current MPXV outbreak exhibited uncommon person-to-person transmission through prolonged exposure or sexual contact with infected individuals (1, 5–7). Despite two vaccines being available for prevention of MPXV infection, insufficient supplies have restricted their accessibility to those in high-risk populations, including immunocompromised individuals and men who have sex with other men. Only two antiviral drugs, tecovirimat and brincidofovir, previously approved by the U.S. FDA for the treatment of smallpox, are available for the treatment of MPXV infection, highlighting the urgent medical need to identify novel antivirals for the treatment of MPXV and other orthopoxvirus infections.

In this study, we first demonstrated the feasibility of using rVACV expressing both fluorescent proteins (Scarlet and GFP) and luciferase (Nluc) to easily identify compounds with inhibitory activity against VACV. Next, we used rVACV Nluc/Scarlet and rVACV Nluc-GFP to test the anti-VACV activity of compounds from the ReFRAME and NPC libraries previously reported to have antiviral activity against RNA viruses (55–59). Using both fluorescent Scarlet or GFP and Nluc readouts, we showed that several of the compounds in the ReFRAME and NPC libraries we tested had inhibitory activity against VACV and that assessing their inhibitory activity using fluorescent-protein (Scarlet or GFP) or luciferase (Nluc) expression resulted in similar EC_50_s and SI values.

Among the compounds in the ReFRAME library, antimycin A and AVN-944 were found to have the strongest inhibitory activity against VACV. It is worth noting that antimycin A has been described to inhibit infection of multiple viruses, including porcine reproductive and respiratory syndrome virus (PRRSV) (64), dengue virus (DENV) (65), IAV and IBV (58), equine encephalitis viruses (EEV), vesicular stomatitis virus (VSV), encephalomyocarditis virus (EMCV), Sendai virus (SeV), and hepatitis C virus (HCV) (66). AVN-944 has also been previously shown to have antiviral activity against both RNA and DNA viruses, likely via reduction of GTP levels in infected cells (67–69). Notably, AVN-944 is currently in phase 1 human clinical trials for cancer treatment (70, 71). Other compounds inhibiting VACV in the ReFRAME library included mycophenolic acid, pyrazofurin, mycophenolate mofetil, azaribine, and brequinar.

The NPC library compounds valinomycin, rotenone, and mubritinib also exhibited potent anti-VACV activity. Other compounds with inhibitory activity against VACV from the NPC library included buparvaquone, narasin, and monensin. Valinomycin has been described to have properties against tumors (72), bacteria (73), fungi (74), and viruses (75, 76), by acting as a potassium ionophore that increases potassium ion permeability of the mitochondrial inner membrane, thereby inhibiting oxidative phosphorylation (77). Monensin is also a selective ionophore that facilitates the transmembrane exchange of sodium ions for protons, which leads to the inhibition of intracellular transport of Golgi apparatus-associated proteins. Monensin has been shown to have anticancer (78) and antiviral properties (79–82), including VACV inhibition (83, 84). Rotenone has been extensively studied and validated as a complex I inhibitor of the mitochondrial electron transport chain (85). Rotenone toxicity in humans is moderate, but it can be used therapeutically at doses posing very low safety risks and has been studied as a potent antiviral (86).

Importantly, six of the compounds from the ReFRAME library and all of the compounds from the NPC library found to have inhibitory activity against VACV were confirmed to also inhibit MPXV. Some of the differences observed in the compounds’ inhibitory activities against VACV and MPXV likely reflect different readouts to assess their inhibitory activity. The ability to use fluorescence or luciferase reporter genes and the lower level of biocontainment required to work with VACV support the use of the bireporter expressing rVACV Scarlet/Nluc and rVACV GFP/Nluc in high throughput screening (HTS) format to identify compounds with inhibitory activity against orthopoxviruses. Importantly, the compounds from the ReFRAME and NPC libraries we tested are FDA approved for use in humans, which should facilitate the process of bringing them into the clinic.

In summary, in this study, we demonstrated that some compounds from the ReFRAME and NPC libraries previously identified to have antiviral activity against different RNA virus families also inhibit VACV and MPXV, demonstrating their potential for the treatment of different viral infections. However, despite having been shown for many years to inhibit viral replication in cell culture systems, some of the identified hit compounds have never been proved to have clinical antiviral activity. Future studies in validated animal models of either VACV or MPXV infection (38, 63) will be necessary to assess the antiviral activity of the compounds *in vivo* and to test their therapeutic potential. It should be noted that the compounds we tested target host factors, which will pose a high genetic barrier to the emergence of viral escape mutants, a common event with antivirals directly targeting a viral protein activity. This is illustrated by tecovirimat resistance, which is the result of mutations in the viral gene encoding the target protein F13 (44). In addition, these results open the possibility of combination therapy using compounds targeting viral factors (e.g., tecovirimat) and host factors (e.g., ReFRAME and NPC compounds) for the treatment of orthopoxvirus infections.

## MATERIALS AND METHODS

### Biosafety.

Experiments with MPXV were performed at biosafety level 3 (BSL3) containment laboratories at Texas Biomedical Research Institute (TX Biomed) and were approved by the Institutional Biosafety Committee (IBC) at TX Biomed. All the researchers involved in studies using MPXV were vaccinated with the modified vaccinia virus Ankara JYNNEOS vaccine.

### Cell lines.

African green monkey kidney fibroblasts (CV-1; ATCC CCL-70) and human adenocarcinoma alveolar basal epithelial cells (A549; ATCC CCL-185) were maintained in Dulbecco’s modified Eagle’s medium (DMEM; Corning) containing 5% fetal bovine serum (FBS) and 1% PSG (100 U/mL penicillin, 100 μg/mL streptomycin, and 2 mM l-glutamine). Cells were grown at 37°C in a 5% CO_2_ atmosphere.

### Viruses.

The two reporter-expressing rVACVs expressing fluorescent proteins (GFP or Scarlet) and luciferase (Nluc), rVACV Nluc/GFP and rVACV Nluc/Scarlet, respectively, were previously described and characterized (54). We used both fluorescent-expressing rVACVs to assess the feasibility of using both GFP and Scarlet coupled with fluorescence microscopy and plate readers to identify compounds with inhibitory activity (54). We selected Nluc because it is a secreted small luciferase that allow us to detect bioluminescence activity in cell culture supernatants and is therefore used in longitudinal studies (54). MPXV USA-2003 (NR-2500) clade II was obtained from the Biodefense and Emerging Infectious (BEI) Resources repository and propagated in CV-1 cells. Virus infections were conducted in DMEM containing 2% FBS and 1% PSG.

### Compounds.

The compounds previously identified to have antiviral activity against RNA viruses in the ReFRAME library were previously described (55–58) and include antimycin A (Sigma-Aldrich, catalog no. A8674), OSU-03012 (AkSci, catalog no. Y0267), mycophenolic acid (AkSci, catalog no. E480), AVN-944 (ADOOQ Bio, catalog no. A13652), 6-azauridine (azauridine; Sigma-Aldrich, catalog no. A1882), pyrazofurin (Sigma-Aldrich, catalog no. SLM1502), mycophenolate mofetil (AkSci, catalog no. J90063), 2′,3′,5′-triacetyl-6-azauridine (azaribine; Sigma-Aldrich, catalog no. T340057), and brequinar sodium salt hydrate (brequinar; Sigma-Aldrich, catalog no. SML0113). The compounds in the NPC library previously shown to have antiviral activity against RNA viruses were also previously described (59) and include azoxystrobin (Sigma-Aldrich, catalog no. 31697), buparvaquone (ArkPharm, catalog no. 88426-33-9), valinomycin (Sigma-Aldrich, catalog no. V0627), narasin sodium salt (narasin; Cayman Chemical, catalog no. 19447), amuvatinib (MedChem Express, catalog no. HY-10206), monensin sodium salt (monensin; Sigma-Aldrich, catalog no. M5273), spautin-1 (MedChem Express, catalog no. HY-12990), tryptanthrin (Sigma-Aldrich, catalog no. SML0310), rotenone (Sigma-Aldrich, catalog no. 45656), and mubritinib (MedChem Express, catalog no. HY-13501). Tecovirimat and 2,2′-bi-1H-benzimidazole (benzimidazole; NSC-67061) were included as positive and negative controls, respectively, in all the assays (41, 87). All compounds were prepared at 10 mM stock solution in dimethyl sulfoxide (DMSO) and kept at −20°C until experimentation. All compounds were diluted in DMEM supplemented with 2% FBS and 1% PSG (infection medium). The maximum concentration of DMSO in all compound preparations was 0.1%, including vehicle control wells.

### Bireporter cell-based assay for the identification of inhibitors.

Confluent monolayers of human A549 cells (96-well plate format, 4 × 10^4^ cells/well, in quadruplicate) were infected with 200 PFU/well of rVACV Nluc/Scarlet or rVACV Nluc/GFP for 1 h at 37°C. After virus absorption, the virus inoculum was removed, and cells were incubated with infection medium containing 3-fold serial dilutions of the indicated compounds (starting concentration of 50 μM for all compounds except for azoxystrobin, buparvaquone, tryptanthrin, and OSU-03012 [starting at 450 μM]; mycophenolic acid, mycophenolate mofetil, and azaribine [starting at 150 μM]; and antimycin A, narasin, monensin, valinomycin and AVN-944 [starting at 1.85 μM]). Mock-infected cells and cells infected in the absence of drug were included as internal controls. At 24 h postinfection (hpi), fluorescence expression (GFP and Scarlet) was visualized using a fluorescence microscope and quantified using a microplate reader (BioTek Synergy). Simultaneously, cell culture supernatants from the same infections were collected and used to measure Nluc expression using the Nano-Glo luciferase substrate (Promega) and the microplate reader (BioTek Synergy). Percent viral infection was determined based on fluorescent (GFP or Scarlet) and Nluc signals, as previously described (58, 88, 89). Experiments were conducted using technical quadruplicates. Microsoft Excel was used to calculate the mean and standard deviation (SD) of viral inhibition from quadruplicate wells (*n* = 4). EC_50_s were determined using sigmoidal dose response curves on GraphPad Prism (version 9).

### FFRA.

Confluent monolayers of human A549 cells (96-well plate format, 4 × 10^4^ cells/well, in quadruplicate) were infected with 200 PFU/well of MPXV for 1 h at 37°C. After virus absorption, the virus inoculum was removed, and cells were incubated with infection medium containing 3-fold serial dilutions (starting concentration of 50 μM for all compounds except for azoxystrobin, buparvaquone, tryptanthrin, and OSU-03012 [starting at 450 μM]; mycophenolic acid, mycophenolate mofetil, and azaribine [starting at 150 μM]; and antimycin A, narasin, monensin, valinomycin and AVN-944 [starting at 1.85 μM]) of the indicated compounds and 1% Avicel (Sigma-Aldrich). Mock-infected cells and cells infected in the absence of drug were included as internal controls. At 24 hpi, cells were fixed in 10% neutral buffered formalin for 24 h and then permeabilized with 0.5% Triton X-100 in phosphate-buffered saline (PBS) for 10 min at room temperature (RT). Then, cells were blocked with 2.5% bovine serum albumin (BSA) in PBS for 1 h, followed by immunostaining with an anti-VACV A33R polyclonal antibody (BEI Resources, NR-628), Vectastain ABC kit, and DAB (3,3′-diaminobenzidine) peroxidase substrate kit (Vector Laboratories), based on the manufacturer’s recommendations. Viral infections were determined based on the number of plaques present in each of the 96-well plates using an ImmunoSpot plate reader, as previously described (57, 58, 90, 91). Similar assays have been described in the literature to assess the antiviral activities of compounds (92, 93). Experiments were conducted using technical quadruplicates. Microsoft Excel was used to calculate the mean and SD of viral inhibition from quadruplicate wells (*n* = 4). Nonlinear regression curves and EC_50_s were determined using sigmoidal dose response curves in GraphPad Prism (version 9).

### Viral titer reduction assays.

Confluent monolayers of human A549 cells (24-well plate format, 2 × 10^5^ cells/well, triplicates) were infected with rVACV Nluc/Scarlet, rVACV Nluc/GFP, or MPXV at an MOI of 0.01 or 3 for 1 h at 37°C. After virus absorption, the virus inoculum was removed, and cells were incubated with infection medium containing 10-fold serial dilutions of tecovirimat (starting concentration of 100 μM). Mock-infected cells and cells infected in the absence of drug were included as internal controls. At 24, 48, and 72 hpi, cell culture supernatant was collected, and Nluc activity was determined by adding Nano-Glo luciferase substrate (Promega) and quantified in a microplate reader (rVACV Nluc/Scarlet or rVACV Nluc/GFP). Viral titers in the cell culture supernatants were determined by plaque assay in CV-1 cells. Briefly, cells were infected with 10-fold serially diluted supernatants for 1 h. After viral adsorption, cells were overlaid with medium containing 1% Avicel (Sigma-Aldrich). Cells were fixed in 10% neutral buffered formalin for 24 h and then permeabilized with 0.5% Triton X-100 in PBS for 10 min at room temperature. Next, cells were blocked with 2.5% BSA in PBS for 1 h and immunostained with an anti-VACV A33R polyclonal antibody (BEI Resources, NR-628) and developed with an anti-rabbit immunoglobulin Vectastain ABC kit and a DAB peroxidase substrate kit (Vector Laboratories), following the manufacturer’s recommendations. Experiments were conducted using technical triplicates. Microsoft Excel was used to calculate the mean and SD of viral inhibition from triplicate wells (*n* = 3).

### Cell viability assays.

MTT (CellTiter 96 nonradioactive cell proliferation assay; Promega) and XTT (cell viability and proliferation assay; Sigma-Aldrich) assays were used to determine A549 cell viability as previously described (58). Briefly, confluent monolayers of human A549 cells (96-well plate format, 4 × 10^4^ cells/well, in quadruplicate) were incubated with 100 μL of infection medium containing 3-fold dilutions of compounds (starting concentration of 50 μM for all compounds except for azoxystrobin, buparvaquone, tryptanthrin, and OSU-03012 [starting at 450 μM]; mycophenolic acid, mycophenolate mofetil, and azaribine [starting at 150 μM]; and antimycin A, narasin, monensin, valinomycin, and AVN-944 [starting at 1.85 μM]) or with 0.1% DMSO as a vehicle control. Then, plates were incubated at 37°C with 5% CO_2_ for 48 h, treated with 15 μL of dye solution for the MTT assay or 50 μL of XTT labeling reagent for the XTT assay, and incubated at 37°C for an additional 4 h. Stop solution was then added to the MTT assay to halt the reaction. Absorbance (570 nm) in each of the wells was measured using a microplate reader (BioTek Synergy). Cell viability was determined as the percentage of values for DMSO vehicle-treated cells. Nonlinear regression curves and the CC_50_ at 48 h were determined using GraphPad Prism software (version 9).

### Statistical analysis.

GraphPad Prism software (version 9) was used for data analysis. CC_50_ and EC_50_s were calculated using sigmoidal dose-response curves, and the selective index (SI) of each compound was determined by dividing the CC_50_ by the EC_50_s. Significance was determined by standard unpaired Student’s *t* test.
