# Correction for Chiem et al., “Identification of *In Vitro* Inhibitors of Monkeypox Replication”

**DOI:** 10.1128/spectrum.03621-23

**Published:** 2023-12-06

**Authors:** Kevin Chiem, Aitor Nogales, Maria Lorenzo, Desarey Morales Vasquez, Yan Xiang, Yogesh K. Gupta, Rafael Blasco, Juan Carlos de la Torre, Luis Martinez-Sobrido

## AUTHOR CORRECTION

Volume 11, no. 4, e04745-22, 2023, https://doi.org/10.1128/spectrum.04745-22. Page 12, Acknowledgments paragraph 2: The second sentence should read as follows. “Research in L.M.-S.’s laboratory is partially funded by grants W81XWH2110103, W81XWH2110095, W81XWH1910496, and W81XWH2210283 from the Department of Defense (DoD) Peer Reviewed Medical Research Program (PRMRP); grants R43AI165089, R01AI161363, R01AI161175, R01AI145332, R01AI142985, R01AI141607, and R21 AI173816 from the National Institute of Health (NIH); the San Antonio Partnership for Precision Therapeutics; the San Antonio Medical Foundation; and the Texas Biomedical Research Institute Forum Foundation.”

